# Research advances in the role of osteoblasts and their derivatives in the development, recurrence, and distant metastasis of malignant bone tumors: a narrative review

**DOI:** 10.1530/EOR-2025-0021

**Published:** 2025-12-05

**Authors:** Xuanhe Huang, Lei Qiang, Yiwei Wang, Zhanyu Meng, Xinyu Dai, Pengfei Zheng

**Affiliations:** Department of Orthopaedics Surgery, Children’s Hospital of Nanjing Medical University, Nanjing, Jiangsu Province, China

**Keywords:** osteoblasts, derivatives, malignant bone tumors, osteoclasts, targeted therapy

## Abstract

- Malignant bone tumors, including primary bone tumors, such as osteosarcoma (OS), Ewing’s sarcoma (ES), and multiple myeloma, and secondary bone tumors from prostate and breast cancers, pose significant mortality risks. Osteoblasts (OBs) and their derivatives play critical roles in the development, recurrence, and metastasis of these tumors.
- OBs promote metastasis-related events, including osteoclast differentiation and proliferation. Their derivatives, including extracellular vesicles and cytokines, modulate bone remodeling and tumor development through various signaling pathways.
- Recent *in vivo* and *in vitro* studies highlight the involvement of OBs in tumor progression, recurrence, and metastasis. Emerging therapies targeting OBs and their derivatives show promise in improving patient outcomes.
- The review emphasizes the importance of understanding the specific roles of OBs and their derivatives in malignant bone tumors. This knowledge can lead to the development of new therapeutic strategies aimed at improving patient survival rates and quality of life.
- Key findings include the regulatory effects of OBs on tumor dormancy, the vicious cycle of bone metastasis, and the potential for targeted therapies to disrupt these processes.
- Future research should focus on developing experimental models that more closely mimic the human tumor microenvironment and integrating multiple signaling pathways to create comprehensive treatment strategies.

## Background

Bone tumors are a rare and heterogeneous group of tumors that occur in the bone, with the prevalence of malignant bone tumors showing an increasing trend (1). Malignant bone tumors include primary bone tumors, such as osteosarcoma (OS), Ewing’s sarcoma (ES), and multiple myeloma (MM), as well as secondary bone tumors originating from other organs, such as the prostate and breast. These tumors pose a significant threat to public health and represent a considerable clinical challenge due to their aggressive nature and the complexities arising from the bone microenvironment.

Primary malignant bone tumors, such as OS and ES, primarily occur in adolescents and children, and their overall treatment outcomes remain suboptimal (2). MM, a plasma cell malignancy is characterized by osteolytic bone lesions in approximately 80% of cases at initial diagnosis (3). Despite treatment, approximately 35% of patients experience disease recurrence and metastasis, consequently resulting in a poor overall 5-year survival rate of less than 25% (4).

Similarly, secondary malignant bone tumors that metastasize from the prostate and breast have a low cure rate and poor prognosis, with approximately 70% of patients developing bone metastases (5). Therefore, further research is warranted to elucidate novel therapeutic targets and develop effective treatment strategies to improve the current situation.

In recent years, an increasing number of studies have indicated that the development, recurrence, and metastasis of these tumors are significantly influenced by the cellular and molecular components of bone, with OBs playing a vital role. OBs, which originate from mesenchymal stromal/stem cells (MSCs), are specialized cells responsible for bone formation and remodeling (6, 7, 8). Recent research has also revealed other critical functions of OBs beyond their traditional roles, particularly their involvement in the pathophysiology of malignant bone tumors (9, 10, 11). OBs and their derivatives can influence the tumor microenvironment through various mechanisms, including the secretion of growth factors, cytokines, and extracellular matrix proteins, as well as their maturation (9, 12, 13, 14). If the biological mechanisms of OBs can be precisely regulated, it is anticipated that new strategies can be developed for malignant bone tumors, effectively curbing their growth and progression.

In this review, we summarize recent progress regarding the role of OBs and their derivatives in the treatment of malignant bone tumors, focusing on their effects on tumor development, recurrence, and metastasis. In addition, we envision the future clinical application of therapeutic regimens based on the regulatory mechanisms of OBs and their derivatives in treating malignant bone tumors.

## Methods

A literature review was conducted using multiple academic databases, including PubMed, MEDLINE, eMedicine, and the National Library of Medicine (NLM). The search was mostly restricted to peer-reviewed articles and reports published between 2020 and 2025. Keywords such as ‘Osteoblasts’, ‘Derivatives’, ‘malignant bone tumors’, ‘osteoclasts’, and ‘targeted therapy’ were employed to optimize the retrieval of relevant studies. Articles were screened based on their relevance to the role of OBs and their derivatives in the development, recurrence, and metastasis of malignant bone tumors. Eligible studies were selected for further analysis.

### Osteoblast differentiation

#### Osteoblast activation and differentiation of regulating mechanism

**Figure 1:**
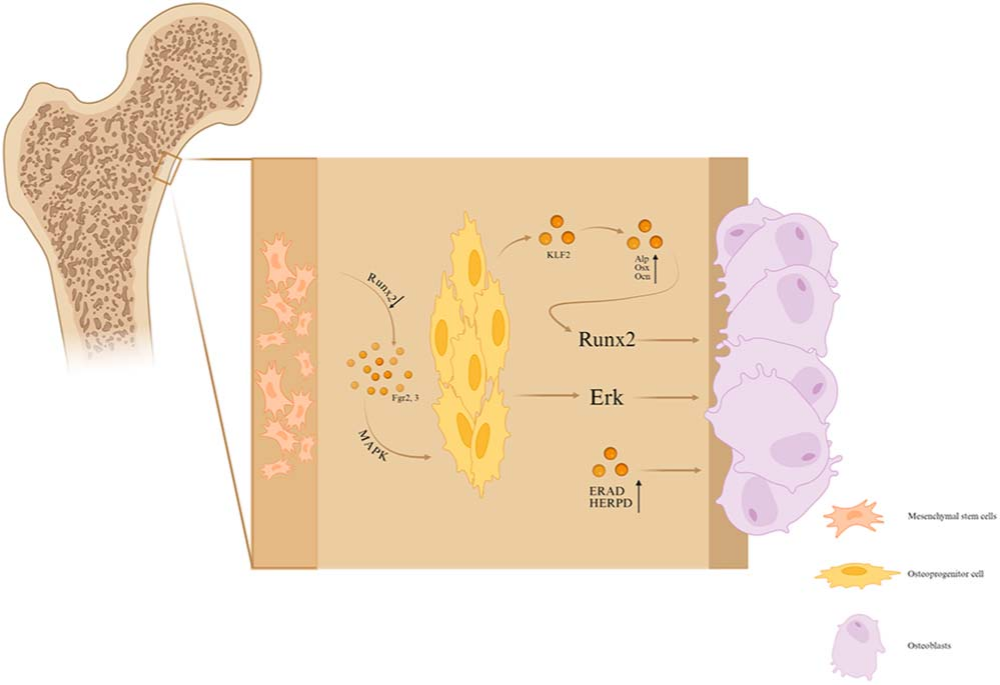
Osteoblast activation and differentiation.

The runt-related transcription factor 2 (RUNX2)/Osterix axis is crucial in the process of OB formation. RUNX2 is essential for OB differentiation and the maturation of cartilage cells. During OB formation, osteoprogenitor cells express RUNX2 and differentiate into preosteoblasts (15). RUNX2 regulates OB progenitor proliferation by inducing fibroblast growth factor receptors 2 (FGFR2) and 3 (FGFR3). FGFR2 and FGFR3 induce proliferation through the mitogen-activated protein kinase (MAPK) signaling pathway (16). Furthermore, RUNX2 expression can be affected by cytokines. Krüppel-like factor 2 (KLF2) is a DNA-binding transcription factor. The overexpression of KLF2 in MC3T3-E1 cells, a preosteoblast line, promotes the expression of osteoblastic differentiation marker genes *Alp*, *Osx*, and *Ocn*, and facilitates mineralization by increasing *Runx2* expression at both the mRNA and protein levels (17). Subsequently, Osterix is expressed in preosteoblasts, leading to their differentiation into OBs. During OB activation, endoplasmic reticulum–associated degradation (ERAD) and homocysteine-inducible endoplasmic reticulum protein with ubiquitin-like domain 1 (HERPUD1) play significant roles. As OB differentiation progresses, both ERAD and proteasomal degradation are activated, leading to an increase in HERPUD1 expression. Consequently, the OB differentiation program is activated by HERPUD1 overexpression (18) (Fig. 1). By elucidating the regulatory mechanisms of osteoblast activation and differentiation, we can not only clarify their biological behavior in malignant bone tumors but also probe deeper into the associated genetic and epigenetic dysregulation, including reprogramming osteoblasts to secrete apoptosis-modulating factors. This approach is essential for targeting the bone microenvironment and developing osteoblast-focused strategies to treat bone tumors.

#### The relationship between the osteoblast and osteoclast

**Figure 2:**
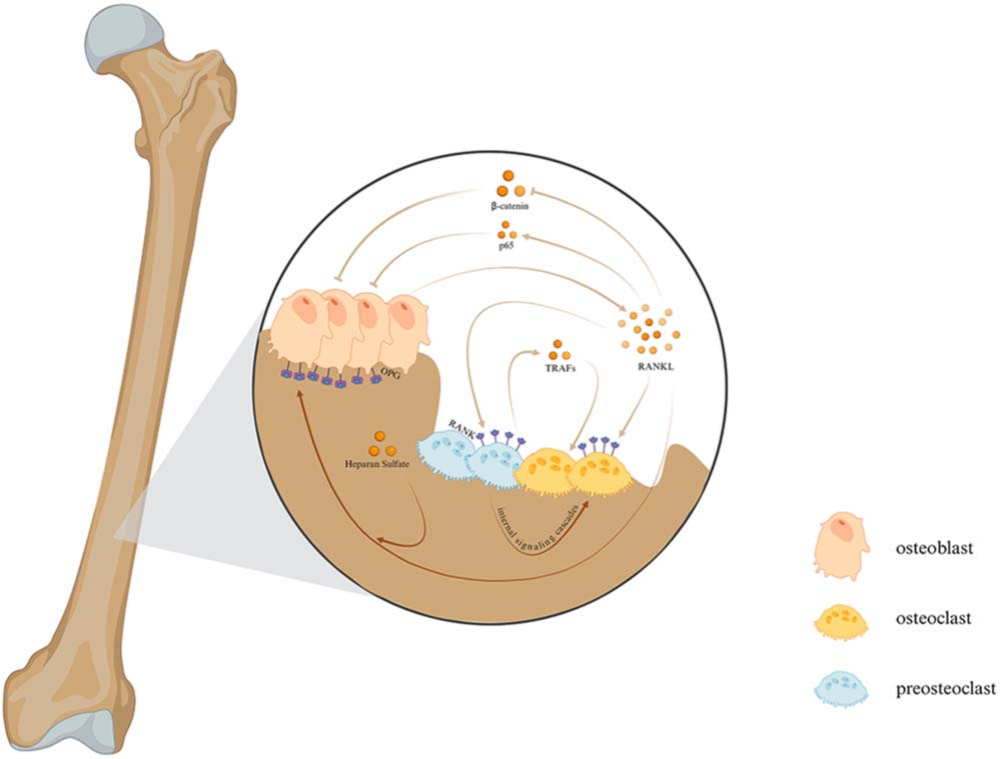
The relationship of osteoblast and osteoclast.

Osteoclasts are terminally differentiated multinucleated cells that originate from the monocyte-macrophage system. Their formation is regulated by various factors, including transcription factors, cytokines, and lymphocytes. Key cytokines involved in this process include macrophage colony-stimulating factor (M-CSF), which is secreted by osteoprogenitor cells, mesenchymal cells, and OBs, and receptor activator of nuclear factor kappa-B ligand (RANKL), which is secreted by OBs and stromal cells (19, 20). Collectively, these factors, most of which are secreted by OBs, promote the activation of transcription factors and the expression of downstream genes in osteoclasts (21, 22). Under physiological conditions, osteoclasts communicate closely with OBs, participate in bone modeling and remodeling, and are involved in calcium homeostasis and bone immunity (23). The regulatory processes and mechanisms involved in the balance between OBs and osteoclasts are highly complex, with receptor activator of RANKL playing a significant role. RANKL, a major cytokine related to tumor necrosis factor, binds to its receptor RANK, located on the surface of preosteoclasts and mature osteoclasts, triggering the differentiation of preosteoclasts into multinucleated, fully functional osteoclasts through tumor necrosis factor receptor-associated factors (TRAFs) and cytoplasmic adapter protein-dependent internal signaling cascades (24). Although RANKL is a critical mediator of osteoclast formation, activation, and survival, it also inhibits osteoblastic differentiation by suppressing β-catenin synthesis and promoting p65 phosphorylation and translocation (25). Osteoprotegerin (OPG), a soluble decoy receptor for RANKL derived from osteoblasts, acts as a key inhibitor of the RANK/RANKL pathway (26). Heparan sulfate anchors secreted OPG at the surface of osteoblast lineage cells, promoting OPG’s binding to membrane-anchored RANKL (27). Thus, OPG inhibits osteoclastogenesis, elevating bone mineral density (26) (Fig. 2). Clarifying the interaction between osteoblasts and osteoclasts is expected to provide new strategies for modulating bone homeostasis and intervening in the development, recurrence, and distant metastasis of malignant bone tumors. Furthermore, the bone organoid models developed based on this knowledge will offer a superior platform for future research on bone-related diseases. These models recapitulate the genetic, phenotypic, and behavioral traits of native tissue while being more cost-effective and requiring shorter maintenance times than traditional cell lines and genetically engineered mouse models.

### Osteoblasts in primary bone tumors

#### Role in tumor development

Wnt signaling has been shown to promote c-Fos-induced OS formation through the actions of the collagen-modifying enzyme Loxl2, as demonstrated in genetically modified mouse models (GEMMs) (28). Inhibiting the secretion of Wnt ligands by inactivating the Wnt-less (Wls) gene in OBs within c-Fos GEMMs reduces Loxl2 expression and slows the progression of OS, both early in development and in therapeutic contexts (28). MM cells overproduce dickkopf-1 (DKK1), which suppresses Wnt signaling in osteoblasts and consequently contributes to myeloma-associated bone lesions (29). The minichromosome maintenance 8 (MCM8)/connective tissue growth factor (CTGF) axis has been identified as a critical participant in the development and progression of OS. As indicators of cell proliferation, the overexpression and knockdown of *MCM8* are significantly associated with the promotion and inhibition of OS cell proliferation *in vitro* and tumor growth *in vivo*. Furthermore, silencing *CTGF*, a potential downstream target of *MCM8*, can mitigate the effects of *MCM8* overexpression on OS development (30). LncRNA H19 disrupts the osteogenic–osteoclastic balance through the Akt/mTOR signaling pathway. H19 deficiency promotes osteoblast differentiation while suppressing osteoclast activity, ultimately leading to significant attenuation of MM cell proliferation (31). In ES, the oncogenic fusion protein EWS-FLI1 – comprising the N-terminus of EWS and the C-terminus of FLI1 – relies on the FLI1 DNA-binding domain (DBD) to drive tumorigenesis. The DBD mediates cooperative binding to contiguous GGAA repeats, forming a nucleoprotein filament essential for oncogenic transformation (8). Emerging evidence identifies ETV6 as a molecular vulnerability in Ewing sarcoma, where it antagonizes EWS::FLI1 function through competitive binding at short GGAA repeat elements (32). In MM plasmas, elevated levels of seven cytokines (ANG1, ENA-78, EGF, PDGF-AA/AB/BB, and TARC) were observed, each demonstrating inhibitory effects on osteogenic differentiation of healthy donor-derived adipocyte-derived MSCs (HD-ASCs), thereby contributing to MM progression (33). Consequently, a crucial future avenue involves applying multi-omics to functionally characterize dysregulated lncRNAs and circRNAs in osteoblasts under tumor influence, ultimately aiming to establish the therapeutic potential of targeting them with oligonucleotide agents.

#### Role in tumor recurrence

**Figure 3:**
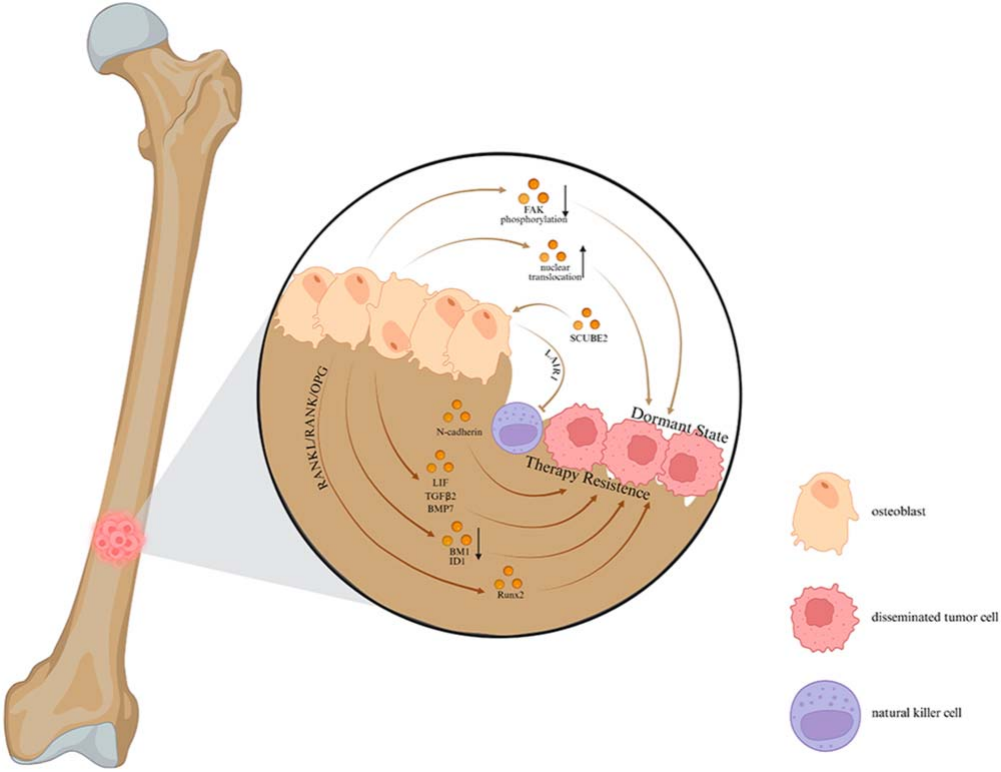
Regulatory effects of osteoblasts and their derivatives on the dormancy of malignant bone tumors.

Approximately 30–40% of patients with localized OS experience recurrence despite aggressive chemotherapy and surgery (34). Furthermore, OS is more likely to recur in patients who undergo non-radical resections (35). Dormant residual cancer cells, such as disseminated tumor cells (DTCs) in the bone marrow (BM), constitute a cellular reservoir for late recurrences, as they can survive therapy in a dormant state and resume proliferation to cause distant relapse, ultimately leading to distant relapse and cancer-associated mortality. The RANKL/RANK/OPG axis, which regulates bone turnover, is activated in DTCs within the BM. By influencing the expression of downstream signals, such as Runx2, this axis can create a bone niche conducive to cancer cell dormancy (36). Moreover, it has been documented that osteoblast-derived factors (e.g., LIF, TGFβ2, BMP7) and adhesion molecules (e.g. N-cadherin) enhance therapy resistance during dormancy (37). Detection of ES-derived circulating EVs, particularly ENO-1+CD81+ and CD99+CD63+ biomarkers, provides a novel tool for precise tumor monitoring, as evidenced by reduced ENO-1+CD63+ levels post-resection and their correlation with tumor burden in Ewing sarcoma-bearing mice (38). Lawson *et al.* demonstrated that the dormancy of MM is a reversible state, which can be ‘switched on’ through contact with bone-lining cells or OBs, while osteoclast-mediated remodeling of the endosteal niche ‘switches it off’ (39) (Fig. 3). Although the role of OBs and their derivatives in cancer recurrence is not fully understood, we propose that spatial transcriptomics and single-cell RNA sequencing will enable us to precisely map the bidirectional signaling network between osteoblasts and dormant tumor cells within the dormant niche.

#### Role in tumor metastasis

**Figure 4:**
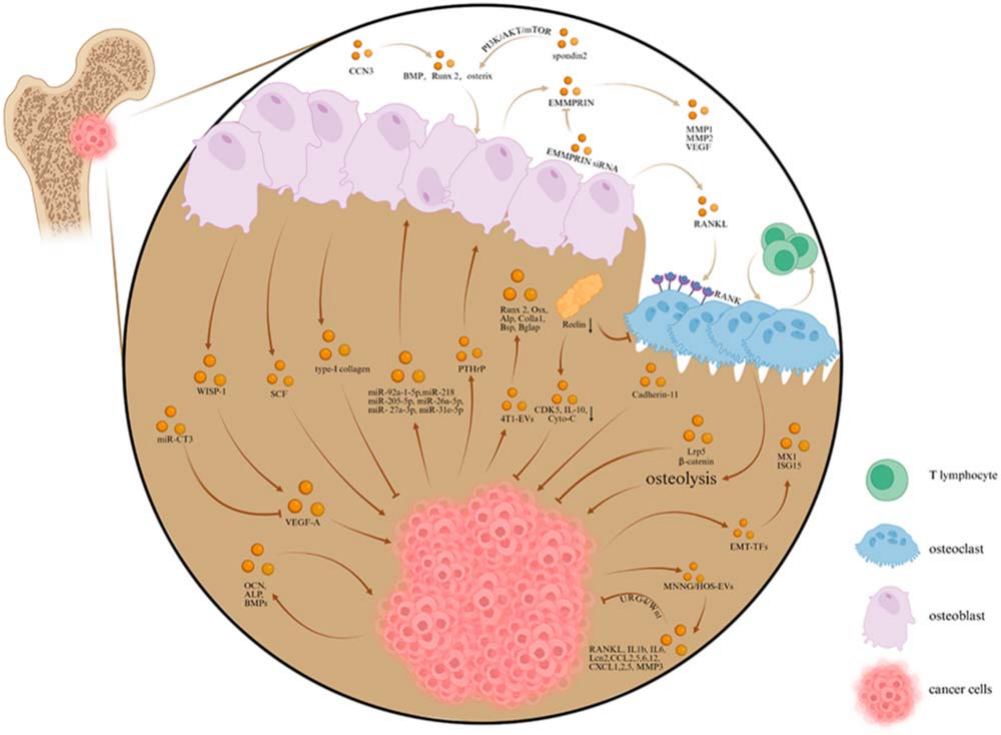
The regulatory effects of osteoblasts and their derivatives on distant metastasis of malignant bone tumors.

Poor prognosis in osteosarcoma patients is closely linked to lung metastasis development, which accounts for nearly all osteosarcoma-related deaths (40). The role of OBs and their derivatives in the distant metastasis of malignant bone tumor cells should not be overlooked. Early studies of bone metastatic cancer observed a phenomenon known as the ‘vicious cycle’, in which the interaction between tumor cells and bone cells accelerates the progression of bone metastatic cancer (41). Tumor cells release substances such as parathyroid hormone-related protein (PTHrP), which stimulates OBs to produce RANKL, activating osteoclasts and resulting in osteolysis. In turn, various factors released by osteolysis further promote tumor development and bone loss (42). Extracellular matrix metalloproteinase-inducing factor (EMMPRIN) is a cell surface glycoprotein expressed in various types of cancer. EMMPRIN promotes a metastatic phenotype by triggering the production of matrix metalloproteinases (MMP1 and MMP2) and vascular endothelial growth factor (VEGF) in both cancer cells and surrounding stromal cells. The expression of MMP2 and VEGF increases following the co-culture of SaOS-2 cells and OBs, and this expression can be suppressed by EMMPRIN siRNA (43). The overexpression of epithelial–mesenchymal transition transcription factors (EMT-TFs) is associated with poor clinical prognosis. We identified two significantly upregulated genes in the EMT pathway: MX1 and ISG15 (13). The EMT-TF ZEB1 can inhibit the differentiation of OBs during normal bone development and in OS cells (44). Furthermore, the overexpression of miR-CT3 can inhibit tumor angiogenesis by decreasing the expression of VEGF-A mRNA and protein, ultimately resulting in restricted metastasis of OS cells (45). The effects of EVs derived from the human OS cell line MNNG/HOS (MNNG/HOS-EVs) on bone-resident cells were evaluated, revealing their anti-osteoblastogenic, pro-inflammatory, and pro-angiogenic properties, which are pivotal to OS metastasis. These EVs reduced the expression of cell cycle and pro-osteoblastogenic genes while increasing the transcriptional expression and protein release of pro-osteoclastogenic and inflammatory cytokines (RANKL, IL-1β, IL-6, and Lcn2), pro-tumoral cytokines (CCL2, CCL5, CCL6, CCL12, CXCL1, CXCL2, and CXCL5), and the metalloproteinase MMP3 (46). The extracellular matrix glycoprotein Reelin promotes MM metastasis. Reduced Reelin expression inhibits osteoclast differentiation while enhancing osteogenesis in LV3-Reln mice. Furthermore, Reelin suppression attenuates extramedullary lesions and prevents splenic immune cell apoptosis through downregulation of CDK5, IL-10, and Cyto-C (47). Cadherin-11 (Cad-11), an osteoblast-enriched adhesion molecule, drives Ewing sarcoma bone metastasis. Its deletion attenuates cellular adhesion/migration and inhibits metastatic progression (48). Notably, EVs derived from OBs effectively suppressed the proliferation of OS cells and promoted their mineralization *in vitro* by targeting the URG4/Wnt signaling pathway (9) (Fig. 4).

Certain physical conditions within the tumor microenvironment can significantly influence bone metastasis in cancer. A novel model of the osteolytic vicious cycle in the BM has been established, indicating that the acidification of the BM microenvironment initiates a process that facilitates tumor expansion and bone destruction. This occurs by inducing, on one hand, the invasion of cancer cells and the attraction of osteoclastic precursors, and on the other hand, the secretion of pro-osteoclastogenic modulators from inflammatory OBs. This process further enhances the vicious cycle of tumor progression and osteolysis, ultimately disrupting the balance of bone remodeling (49). The immune microenvironment in the BM plays a crucial role in the metastasis of OS. It has been documented that a hypo-proliferative subpopulation initiates metastasis by persistently producing IL6 and CXCL8 in response to lung epithelial cell-derived IL1α (50). Exosomes derived from bone tumors can influence bone cell behavior and hijack critical physiological pathways to promote survival and proliferation, thereby creating a permissive microenvironment that favors tumor cell homing. Studies have shown that exosomes from pulmonary osteosarcoma metastases initiate OS secondary metastasis by delivering the miR-194/215 cluster to suppress myristoylated alanine-rich C-kinase substrate (MARCKS) (51). However, the role of osteoblast-derived exosomes in primary bone tumors remains elusive. Consequently, in-depth investigation of these exosomes and the precise regulation of their secretion represent a promising therapeutic strategy for inhibiting bone tumor metastasis.

### Osteoblasts in secondary bone tumors

#### Role in tumor development

Secondary tumors from other organs represent a significant source of malignant bone tumors, with bone metastases from prostate cancer and breast cancer being the most common and lethal. Recent studies have shown that RNF41 induces noncanonical polyubiquitination of MYO1C, enhancing its stability and promoting actin remodeling, which facilitates the bone metastasis of prostate cancer (52). Furthermore, by employing machine learning, gene ontology (GO), Kyoto Encyclopedia of Genes and Genomes (KEGG), gene set enrichment analysis (GSEA), single-cell analysis, and receiver operating characteristic methods, researchers have identified seven hub genes associated with PCa bone metastasis: *APOC1*, *CFH*, *NUSAP1*, *LGALS1*, *NR4A2*, *ADRB2*, and *ZNF331* (53). Compared to adjacent normal tissues, *BMP8A* is upregulated in triple-negative breast cancer (TNBC) and is associated with a poor distant metastasis-free survival rate (DMFS) (54). In addition, both exosomal miR-19a and Integrin-Binding Sialoprotein (IBSP) are significantly upregulated, with IBSP attracting osteoclasts and creating an osteoclast-enriched environment in the bone, thereby facilitating the delivery of exosomal miR-19a to osteoclasts to induce osteolysis (55).

Prostate cancer (PCa) is the second most prevalent malignancy among men worldwide (56). Distant metastasis primarily occurs in advanced stages of PCa and is the leading cause of mortality associated with this disease. The median survival rate for patients with bone metastasis is less than 3 years, and the average 5-year survival rate is only 3% (57). The process of bone metastasis in PCa is complex, involving the circulation of tumor cells into the BM, where they may enter a dormant state before reactivation and proliferation occur. Similar to OS metastasis, exosomes play a significant role in the development of bone metastasis in PCa. Jun-qi Luo *et al.* demonstrated that exosomal phosphoglycerate mutase 1 (PGAM1) promotes angiogenesis in metastatic PCa patients through both *in vivo* and *in vitro* experiments. In addition, exosomal PGAM1 binds to γ-actin (ACTG1), facilitating podosome formation and neovascular sprouting in human umbilical vein endothelial cells (HUVECs), ultimately contributing to the development of bone metastasis in PCa (58).

Breast cancer (BCa) is the second most common form of cancer, following lung cancer, with over 1.3 million women diagnosed worldwide each year (59). Metastatic lesions are the leading cause of death among patients, with the bone being a common site of metastasis (60). Exosomes play a crucial role in breast cancer development, metastatic lesions, and therapeutic resistance. MicroRNA (miRNA) encapsulated in exosomes plays an important role in the bone metastasis of breast cancer cells. It has been found that triple-negative BCa EVs deliver a specific miRNA combination (miR-185-5p, miR-652-5p, and miR-1246) to normal fibroblasts, reprogramming them into cancer-associated fibroblasts. This process is mediated through the direct targeting of POZ/BTB and AT hook containing zinc finger 1 (PATZ1) by miR-185-5p and miR-652-5p, whose downregulation enhances the secretory profile of these activated fibroblasts (61).

Utilizing both the TCGA-BRCA and E-MTAB-4003 databases, differentially expressed genes were systematically analyzed. Researchers have found that *DPP9*, *FAS*, *ZNF519*, *RPP14*, and *FAU* are actively involved in the adaptive colonization of bone metastatic breast cancer cells (62). In addition, the function of growth differentiation factor-15 (GDF15) was evaluated. GDF15 promotes OBs’ function and facilitates the growth of PCa in bone through the osteoblastic production of CCL2 and RANKL, as well as the recruitment of osteomacs (12). Furthermore, a Notch3-MMP-3 axis in human prostate cancer bone metastases was identified as contributing to osteoblastic lesion formation by inhibiting osteoclast differentiation and facilitating osteoblastogenesis, thereby revealing a new approach to manipulating the tumor microenvironment in bone metastases (63).

Before bone colonization, immune cells activated by primary breast tumor cells actively modify the bone microenvironment (BME), disrupting the complex and tightly regulated signaling network maintained by OBs and osteoclasts. A recent study demonstrated that CD8+ T cell phenotypes derived from the 67NR+ tumor contribute to bone homeostasis and/or the control of 4T1 breast tumor pre-metastatic disease, interfering with the activities of osteoclasts and OBs within the BM (64). The expression of *Runx2*, osterix (*Osx*), alkaline phosphatase (*Alp*), collagen type I (*Col1a1*), bone sialoprotein (*Bsp*), and osteocalcin (*Bglap*) can be decreased by EVs derived from 4T1 bone metastatic mouse mammary tumor cells (4T1-EVs) during the late stage of OB differentiation (65). This finding provides new insight into the pathological impact of osteolytic bone metastasis. Circulating miR-218 is closely associated with breast cancer bone metastasis. Although OBs actively contribute to cancer progression by facilitating a destructive bone cycle, a recent study has revealed a counterintuitive mechanism in cancer-associated bone metastasis. Specifically, the overexpression of *Lrp5* and β-catenin in Wnt signaling renders their conditioned medium (CM) tumor-suppressive and bone-protective (66).

Skeletal metastasis is the leading cause of morbidity and mortality in patients with prostate cancer. Once tumor cells metastasize to the bone, the survival rate of patients drops to less than 30% (67). Foster *et al.* discovered that the stem cell factor (SCF, Kit Ligand) derived from OBs plays a crucial role in the composition of BM-derived progenitor cells and the formation of pre-metastatic niches (68). Activated OBs stimulate the progression of prostate cancer in the bone. CCN3 (nephroblastoma-overexpressed), a cysteine-rich protein belonging to the CCN family, enhances the expression of bone morphogenetic protein (BMP), RUNX2, and osterix through GSK3β and β-catenin signaling (69). The expression of Runx2 and osterix can also be promoted by spondin 2, a diagnostic marker specific to prostate cancer. This process is closely related to the activation of the PI3K/AKT/mTOR pathway (14). EVs derived from PCa cells play a crucial role in reprogramming OBs and supporting niche formation before metastasis. EVs containing nuclear-enriched abundant transcript 1 (NEAT1) from PCa cells increase the expression of Runx2, promoting the osteogenesis of human BM-derived mesenchymal stem cells (hBMSCs) by competitively binding to miR-205-5p via the SFPQ/PTBP2 axis, which induces osteoblastic-type bone metastasis (70). In addition, miR-92a-1-5p, the most highly expressed miRNA shuttled in EVs, downregulates type I collagen expression by directly targeting COL1A1, a primary antibody, thereby promoting the differentiation of osteoclasts and inhibiting the differentiation of OBs (71). In addition, miR-26a-5p, miR-27a-3p, and miR-30e-5p are involved in the suppression of BMP-2-induced osteogenesis *in vivo*, specifically in a murine PCa cell line, highlighting the essential role of these EV-derived miRNAs in the PCa-mediated suppression of OB activity (72). Upon entering the niche, PCa cells acquire an osteoblast-like phenotype by releasing molecules such as osteocalcin (OCN), alkaline phosphatase (ALP), and bone morphogenetic proteins (BMPs) that are involved in osteocyte differentiation and maintenance. This process leads to tumor growth, bone destruction, and the activation of a vicious cycle of bone remodeling (73) (Fig. 4). The accumulated evidence solidifies that the overarching goal for future research entails a deep investigation into the EV-mediated crosstalk between tumor cells and osteoblasts, coupled with the development of bidirectional strategies to reprogram EV cargo and function, inhibiting pro-tumorigenic EVs while promoting anti-tumorigenic EV activity to ultimately suppress distant metastasis.

#### Role in tumor recurrence

The role of the immune system in the maintenance and escape from prostate cancer (PCa) dormancy is relatively underexplored. However, significant effects have been identified. The Wnt/β-catenin pathway acts as a key orchestrator of the immunosuppressive tumor microenvironment. Insulin-like growth factor 1 (IGF1) and asporin (ASPN) drive prostate cancer development by activating Wnt/β-catenin signaling in prostatic basal progenitors, thereby inducing oncogenic transformation (74, 75).Besides, Wnts promote immunosuppression and indirect oncogenesis by recruiting M2-polarized TAMs that inhibit CD8^+^ T-cell activity (76). Therefore, inhibition of the Wnt/β-catenin pathway may potentially sustain a dormant state in prostate cancer cells. Owen *et al.* demonstrated that tumor cell-intrinsic type I interferon, as opposed to microenvironmental factors, is critical for maintaining PCa dormancy (77). Once the dormancy of tumor cells is disrupted, the remaining tumor cells can rapidly proliferate, leading to the recurrence of malignant bone tumors.

Osteoblast-like cells derived from humans seemed to bring about a dormant phenotype by reducing the expression of migration- and proliferation-related proteins, such as BMI1 and ID1, in primary breast cancer 3384T cells (78). In addition, SCUBE2-induced osteoblast differentiation facilitates bone relapse of tumors by depositing collagen, which suppresses natural killer cells through the inhibitory LAIR1 signaling pathway (79). Furthermore, OBs play a crucial role in inducing prostate cancer cell dormancy by inhibiting mitochondrial-related biological processes, where focal adhesion kinase phosphorylation is decreased and nuclear translocation is increased (80) (Fig. 3). We propose that future research should focus on elucidating the mechanisms by which osteoblasts modulate the function of CD8+ T cells and other immune cells, and how this interaction ultimately affects tumor immune surveillance and escape. Consequently, investigating the potential synergistic effects of combining osteoblast-targeted therapies with immune checkpoint inhibitors represents a highly valuable translational research direction.

### Treatment options targeting osteoblasts

#### Tumor recurrence

The recurrence of malignant bone tumors involves a series of events, including drug resistance in tumor cells and the reactivation of dormant tumor cells. In the BM microenvironment, where breast cancer DTCs can remain dormant for decades, NG2+/Nestin+ MSCs can induce dormancy in breast cancer DTCs by producing transforming growth factor beta 2 (TGFβ2) and bone morphogenetic protein 7 (BMP7). This process activates a quiescence pathway dependent on TGFβ receptor III (TGFBRIII) and bone morphogenetic protein receptor II (BMPRII), which, via p38 mitogen-activated protein kinase (p38MAPK), leads to the induction of *p27* (81). Furthermore, the TGFβRIII-p38MAPK-pS249/pT252-retinoblastoma (RB) signaling pathway has been identified as a mechanism by which DTCs are induced into a dormant state. Further studies have indicated that aging may lead to reactivation from dormancy and subsequent BCa bone relapse. Furthermore, it has been demonstrated that osteoblastic PKD1/CREB1/GAS6 signaling regulates the cellular dormancy of prostate cancer. Mechanistically, protein kinase D1 (PKD1) in OBs induces cellular dormancy by activating *CREB1*, which promotes the expression and secretion of growth arrest-specific 6 (GAS6) (10). Therefore, maintaining a delicate balance between the TME and dormant tumor cells, as well as addressing the drug resistance of tumors, is critical for managing the recurrence of malignant bone tumors.

#### Tumor metastasis

In the earlier sections of this article, we discussed the role of OBs and their derivatives in the metastasis of malignant bone tumors. Here, we briefly summarize the main mechanisms involved. The vicious cycle, closely associated with the RANKL/RANK axis, RUNX2 signaling, and Wnt signaling, plays a crucial role in bone metastasis. Furthermore, this cycle can be influenced by various factors, including EVs and cytokines. In addition, myeloid-derived suppressor cells (MDSCs), MSCs, extracellular matrix metalloproteinases inducing factor (EMMPRIN) on the cell surface, MMP1, MMP2, and vascular endothelial growth factor (VEGF) can affect the behavior of OBs and the interaction between metastatic tumor cells and the tumor microenvironment (TME), thereby influencing the metastasis of bone tumors (43). It has also been found that osterix (*Osx*), a zinc finger-containing transcription factor, facilitates the bone metastasis of breast cancer by upregulating the expression of a series of genes that contribute to various steps in the metastatic cascade (82). In addition, growth differentiation factor 11 (GDF11), CD151, PAFAH1B2, and YTHDF2, which are significantly differentially expressed genes, play a vital role in the predisposition of breast cancer metastasis to bone, which is associated with homing, immune escape, angiogenesis, and factors involved in both osteoblastogenesis and osteoclastogenesis (62).

#### Currently applied osteoblast-related treatment

One essential measure to enhance the development of more effective treatments for bone metastases is the creation of *in vitro* models that accurately replicate the native bone tumor microenvironment. In addition to identifying the critical mechanisms regulating the recurrence and metastasis of malignant bone tumors, several drugs are currently being studied in the laboratory and show promising potential. Furthermore, some drugs have been utilized in clinical practice and have yielded encouraging results. OBs reportedly secrete the adrenal androgen precursor dehydroepiandrosterone (DHEA), which does not activate androgen receptors but promotes cancer progression and metastasis (83). This suggests that the development of antibodies against DHEA may serve as a promising strategy to inhibit cancer progression and metastasis. The CDK4/6 inhibitors (CDKi) palbociclib, ribociclib, and abemaciclib have been approved for use in combination with anti-estrogen therapy for the treatment of advanced and/or metastatic hormone receptor-positive/HER2 – neu-negative breast cancer (84). In contrast, Abiraterone, a selective inhibitor of androgen biosynthesis, has been approved for the treatment of metastatic prostate cancer, both castration-resistant and castration-sensitive, by modulating multiple OB proliferative signals (85). Metformin has been shown to effectively reduce tumor growth by inhibiting cancer cell viability, blocking cancer cell-induced osteoblastic RANKL/RANK/c-Fos/NFATC1 signaling, which further activates osteoclastogenesis, and directly reducing osteoclast differentiation (86). Procoxacin (Pro), which sensitively reflects the intensity of prostate cancer (PCa) and OB interaction, inhibits PCa-induced osteoblastic changes without affecting the viability of OBs or PCa cells. In addition, it directly kills OCs or suppresses osteoclastic functions at very low concentrations by disrupting the feedback loop of the TGF-β/C-Raf/MAPK pathway (87). Deletion of src homology-2 containing protein tyrosine phosphatase (SHP2) in Bglap+ OBs has been shown to inhibit osteogenic differentiation via RUNX2/Osterix7 signaling (88). Therefore, treatment strategies targeting SHP2 hold promise for future applications. The combination of denosumab monoclonal antibody (DNmb), which binds to RANKL, and docetaxel (TXT), an anticancer drug encapsulated in sustained-release biodegradable nanoparticles (TXT-NPs) localized in BM, has shown significant effectiveness in treating bone metastasis without compromising the activity of OBs and osteoclasts (89). Glycine 34-to-tryptophan (G34W) substitutions in histone H3.3 occur in approximately 90% of giant cell tumors of bone (GCT), which are essential for tumor formation, the pathological recruitment of giant osteoclasts, and subsequent bone destruction (90). This finding supports the feasibility of applying Clustered Regularly Interspaced Short Palindromic Repeats with CRISPR-associated protein 9 (CRISPR–Cas9) technology in the context of metastatic bone tumors. In addition, a new subpopulation of OBs, termed ‘educated’ OBs (EOs), has been identified in the bone tumor microenvironment. These EOs reduce osteoclast formation and bone resorption, indicating a protective role in the skeleton (91). The activation and differentiation of OBs can be regulated by various growth factors and cytokines, including bone morphogenetic protein 2 (BMP2) and Wnt signaling. Classical Wnt/β-catenin signaling promotes bone deposition by facilitating the binding of Wnt ligands to membrane-bound frizzled receptors and Lrp coreceptors on OBs (92). In addition, Wnt signaling enhances bone formation through OB activation by reducing the expression of Dickkopf-related protein 1 (DKK1) and sclerostin (93). Because of the limited vascularity of bone tissue, which contributes to inefficient drug delivery and low local drug concentrations, the use of the HA-BSA-PTX nanodrug delivery system, a ternary nanoscale biomaterial/antitumor drug complex comprising hydroxyapatite (HA), bovine serum albumin (BSA), and paclitaxel (PTX), offers higher delivery efficiency, sustained drug release, and exhibits antitumor and osteogenic effects, making it a promising agent for OS adjuvant therapy (94).

However, given the pivotal physiological roles of osteoblasts, the safety of their targeted therapies requires careful evaluation. On one hand, excessive activation of osteoblasts may lead to pathological bone hyperplasia, adversely affecting patients’ quality of life. Conversely, therapeutic strategies that inhibit osteoblast function or induce their apoptosis can result in bone loss and reduced bone density, thereby significantly increasing the risk of pathological fractures. This risk is particularly critical for patients with primary bone tumors or advanced cancer with bone metastases. Consequently, developing treatment regimens must strike a balance between efficacy and skeletal safety.

## Conclusion

In this review, we thoroughly and systematically elaborate on the pivotal regulatory roles of OBs and their derivatives in the development, recurrence, and distant metastasis of malignant bone tumors. Recent advances in research regarding the role of OBs and their derivatives have provided new insights into treatment strategies targeting these cells, indicating a promising future for improved survival and more favorable prognoses. Significant progress has been made in the research and development of drugs targeting OBs, with some currently in clinical trials, and additional candidate drugs demonstrating encouraging efficacy in laboratory settings. These drugs aim to inhibit the recurrence and metastasis of malignant bone tumors by precisely interfering with the specific functions of OBs. The combination of molecularly targeted drugs with traditional treatments, such as surgery, radiotherapy, and chemotherapy, offers unprecedented therapeutic advantages and greater hope for patients with metastatic bone tumors.

Despite rapid advancements in research, several limitations persist in current studies. The primary issue is that experimental models, including cell lines and animal models, cannot fully replicate the complex tumor microenvironment of the human body. This limitation contributes to the disparity between the performance of certain drugs in clinical trials and the expected outcomes. Consequently, developing experimental models, such as bone organoid and humanized models, that more closely resemble the human environment and reduce the use of laboratory animals has become an urgent scientific challenge. In addition, the heterogeneity of the tumor microenvironment and its intricate internal signaling networks impose greater demands on systematic research. Future investigations should focus on integrating the mechanisms of multiple signaling pathways to establish a comprehensive and systematic treatment strategy. This approach not only necessitates further exploration of the interactions between signaling pathways and regulatory networks, but also requires identifying targets for more coordinated interventions to achieve more precise tumor treatment.

## ICMJE Statement of Interest

The authors declare that there is no conflict of interest that could be perceived as prejudicing the impartiality of the work reported.

## Funding Statement

This work did not receive any specific grant from any funding agency in the public, commercial, or not-for-profit sector.

## Author contribution statement

XH was responsible for conceptualization, writing the original draft, data curation, investigation, formal analysis, methodology, resources, validation, and visualization. LQ was responsible for conceptualization, project administration, writing review and editing, and supervision. YW was responsible for conceptualization, formal analysis, project administration, writing review and editing, and supervision. ZM was responsible for conceptualization. XD was responsible for conceptualization. PZ was responsible for conceptualization, methodology, funding acquisition, formal analysis, project administration, supervision, and writing review and editing. All authors listed have made a substantial, direct, and intellectual contribution to the work and approved it for publication.
